# Is Banding an Acceptable Treatment for Varices that have not Bled (Prophylaxis)?

**DOI:** 10.1155/1999/42608

**Published:** 1999-04

**Authors:** Gregory V. Stiegmann

**Affiliations:** University of Colorado Health Science Center 4200 East 9th Ave. Denver Colorado 80262 USA


*HPB Surgery*, 1999, Vol. 11, pp. 195–206
Reprints available directly from the publisher
Photocopying permitted by license only

© 1999 OPA (Overseas Publishers Association) N.V.
Published by license under
the Harwood Academic Publishers imprint,
part of The Gordon and Breach Publishing Group.
Printed in Malaysia.

### HPB INTERNATIONAL

**EDITORIAL & ABSTRACTING SERVICE**

**JOHN TERBLANCHE EDITOR**

Department of Surgery,
Medical School · Observatory 7925
Cape Town · South Africa
Telephone: 27-21-406-6204
Telefax 27-21-448-6461
E-mail: jterblan@uctgsh1.uct.ac.za

### Is Banding an Acceptable Treatment for Varices that have not Bled (Prophylaxis)?

### ABSTRACT

*Lay, C.-S., Tsai, Y.-T., Teg, C.-Y., Shyu, W.-S., Guo, W.-S., Wu, K.-L. and Lo, K.-J. (1997) Endoscopic variceal ligation in prophylaxis of first variceal bleeding in ciorrhotic patients with high-risk esophageal varices. Hepatology; 25, 1346–1350.*

**To determine the efficacy of endoscopic variceal ligation (EVL) in prophylaxis on the rate of first esophageal variceal bleeding, we conducted a prospective, randomized trial in 126 cirrhotic patients with no history of previous upper gastrointestinal bleeding and with esophageal varices endoscopically judged to be at high risk of hemorrhage. The endpoints of the study were bleeding and death. Life-table curves showed that prophylactic EVL significantly diminished the rate of variceal hemorrhage (12/62 [19%] *vs.* 38/64 [60%]; $P = 0.0001$) and overall mortality (17/62 [28%] *vs.* 37/64 [58%]; $P = 0.0021$). The 2 year cumulative bleeding rate was 19% (12/62) in the EVL group and 60% (38/64) in the control group. The 2 year cumulative mortality rate was 28% (17/62) in the EVL group and 58% (37/64) in the control group. Comparison of Kaplan-Meier estimates of the time to death of both groups showed significantly lower mortality in the ligation group ($P = 0.001$). Patients undergoing EVL had few treatment failures and died mainly of hepatic failure. The lower risk in the EVL group was attributed to a rapid reduction of variceal size. Prophylactic EVL was more efficient in preventing first bleeding in patients with good condition (Child A) than in those with decompensated disease (Child B and C). We conclude that prophylactic EVL can decrease the incidence of first variceal bleeding and death over a period of 2 years in cirrhotic patients with high-risk esophageal varices. (*Hepatology*, 1997; 25, 1346–1350).**

*Keywords:* Variceal band ligation, variceal banding, portal hypertension, variceal prophylaxis

### PAPER DISCUSSION

Endoscopic treatment as prophylaxis against a first variceal bleed (primary prophylaxis) is not widely accepted outside of Japan. The most studied prophylactic endoscopic treatment is sclerotherapy. Western patients judged to be at high risk for a first variceal bleed and treated with endoscopic sclerotherapy fared better than those who had no treatment in some trials [1] and had outcomes equal or worse than those who received no treatment in the others [2]. Western physicians, with few exceptions, have moved away from further consideration of endoscopic sclerotherapy as a viable method for prevention of a first variceal bleed and have focused instead on drug therapy.

Lay *et al.*, from Taiwan, report results from the second trial of endoscopic band ligation as primary prophylaxis [3]. Patients with cirrhosis and portal hypertension from hepatitis B were evaluated using criteria developed by the Japanese Research Society for Portal Hypertension and Beppu to assess risk of hemorrhage [4, 5]. Those at high risk for a first variceal bleed by these criteria were randomized to endoscopic band ligation or no treatment. Endoscopic ligation was performed using the original single fire ligating device delivered *via* an endoscopic overtube. As in the first trial, performed by Sarin *et al.*, in India, patients treated with endoscopic ligation had significantly less chance of experiencing a first variceal bleed, few complications related to endoscopic treatment, and less chance of dying (statistically less in the current study; a trend in Sarin's trial) [6].

Is it time for those in the West to rethink the role of the endoscopist in prophylaxis against a first variceal bleed? There is little question that endoscopic band ligation is associated with fewer complications than sclerotherapy. Band ligation also results in faster eradication of varices (fewer treatments to eradicate) although it is hard to understand how this advantage could prevent bleeding in patients who have not yet bled. On the other hand, in most trials which compared ligation with sclerotherapy for prevention of recurrent variceal hemorrhage, ligation was associated with less chance for further bleeding than sclerotherapy [7]. Perhaps the occasional deep sclerosant induced ulcer, or what appears to be greater local inflammatory response elicited by sclerosant injection, is responsible for a higher risk of hemorrhage with sclerotherapy treatment. It is true that patients treated with band ligation have a greater incidence of recurrence of varices, once initial eradication of varices has been achieved, than those treated with sclerotherapy; however, with proper surveillance and repeat treatment if needed, the practical consequences are few. The biggest single advantage for band ligation over sclerotherapy, in my opinion, is its relative lack of operator dependence. Once the endoscopic ligator is delivered to the target area, the operator does little but select the target, push the endoscope suction button, and pull a string. The resulting tissue effects are homogeneous, reproducible, and are confined to the mucosa and submucosa [8]. The only operator critical component of traditional band ligation is overtube placement. In most large trials of secondary prophylaxis, as in the two trials of primary prophylaxis, overtube related problems are uncommon. Scattered reports of overtube induced mucosal injury or perforation have focused attention on this potential disadvantage of band ligation. Introduction of multiple-fire ligating devices has eliminated the need for an overtube, albeit at significantly increased cost.

Safety and reproducibility of results are key requirements for successful treatment aimed at prevention of a first variceal bleed. Only about one in three patients with cirrhosis and varices ever bleed [9]. Unnecessary treatment of two thirds, particularly with an invasive treatment such as endoscopy, is difficult to advocate. Only if identification of those patients at high risk for a first bleed can be consistently achieved (60% incidence of hemorrhage in the Lay study control group) can endoscopic measured be justified.

Pharmacological treatment with beta adrenergic receptor blocking agents has been shown effective in decreasing the risk of a first variceal bleed and probably reduces mortality when compared with no treatment [10]. Drug therapy is based on sound physiologic principles, is relatively inexpensive, has the intrinsic appeal of being completely operator independent, (although it is very patient dependent) and is administrable by anyone who is authorized to write a prescription. Addition of isosorbide mononitrate to the prophylactic beta-blockade regimen has been shown, in one trial, to result in even greater protection [11]. This study and results from trials examining the two drug regimen for secondary prophylaxis argue for a two drug regimen in future pharmacological trials of primary prophylaxis.

The two published trials which demonstrate efficacy of band ligation for primary prophylaxis do not yet justify a conclusion that this treatment should be recommended for patients with cirrhosis and varices which have not bled – even for those at high risk of a first bleed. It is safe to conclude that band ligation has the potential to become established as a safe and effective method for prevention of a first variceal bleed, but further confirmation is needed. Future trials of endoscopic prophylaxis should avoid an untreated control arm and compare band ligation with a one or two drug regimen in patients who are determined to be at high risk for a first bleed.

I predict that drug therapy and endoscopic band ligation will eventually be found equally effective for prevention of a first variceal bleed. Patients themselves will determine which regimen is superior. Those who have capacity for compliance with a daily, lifelong, drug regimen will probably be best treated with medications. Others who are less compliant, are intolerant of the medications, or who desire a finite period of treatment, will be best served with the endoscopic solution.

### *References*

[1] Paquet K. J., Klein, C. P. and Gad, H. A. (1994). Prophylactic sclerotherapy for esophageal varices in high-risk cirrhotic patients selected by endoscopic and hemodynamic criteria: A randomized, single-center, controlled trial. *Endoscopy*, **26**, 734–40.

[2] The Veterans Affairs Cooperative Variceal Sclerotherapy Group. (1991). Prophylactic sclerotherapy for esophageal varices in men with alcoholic liver disease: A randomized, single-blind, multicenter trial. *New Eng. J. Med.*, **324**, 1779–84.

[3] Lay, C.-S., Tsai, Y.-T., Teg, C. Y., Shyu, W. S., Guo, W. S., Wu, K. L. and Lo, K. J. (1997). Is banding an acceptable treatment for varices that have not bled (prophylaxis)? *Hepatology*, **25**, 1346–50.

[4] Beppu, K., Inokuchi, K., Koyanagi, N. *et al.* (1981). Prediction of variceal hemorrhage by esophageal endoscopy. *Gastrointest. Endosc.*, **27**, 213–8.

[5] Japanse Research Society for Portal Hypertension. (1980). The general rules for recording endoscopic findings on esophageal varices. *Jpn. J. Surg.*, **10**, 84–91.

[6] Sarin, S. K. G. R., Jain, A. K. and Sundaram, K. R. (1996). A randomized controlled trial of endoscopic variceal band ligation for primary prophylaxis of variceal bleeding. *Eur. J. Gastroenterol. Hepatol.*, **8**, 337–42.

[7] Laine, L. and Cook, D. (1995). Endoscopic ligation compared with sclerotherapy for treatment of esophageal variceal bleeding: a Meta analysis. *Ann. Int. Med.*, **123**, 280–7.

[8] Stiegmann, G. V., Sun, J. H. and Hammond, W. S. (1988). Results of experimental endoscopic esophageal varix ligation. *Am. Surg.*, **54**, 105–8.

[9] Burroughs, A. K., D'Heygere, F. and McIntyre, N. (1986). Pitfalls in studies of prophylactic therapy for variceal bleeding in cirrhotics. *Hepatology*, **6**, 1407–13.

[10] Pagliaro, L. D. G., Sorensen, A., Lebrec, D., Burroughs, A. K., Morabito, A., Tine, F., Politi, F. and Traina, M. (1992). Prevention of first bleeding in cirrhosis: A meta-analysis of randomized trials of nonsurgical treatment. *Ann. Int. Med.*, **117**, 59–70.

[11] Merkel, C. M. R., Enzo, E., Donada, C., Cavallarin, G., Torboli, P., Amodio, P., Sebastianelli, G., Sacardoti, D., Felder, M., Mazzaro, C., Beltrame, P. and Gatta, A. (1996). Randomised trial of nadolol alone or with isosorbide mononitrate for primary prophylaxis of variceal bleeding incirrhosis. Gruppo-Trivento per L'ipertensione portale. *Lancet*, **348**, 1677–81.

Gregory V. Stiegmann, MD
Professor of Surgery
University of Colorado Health Science Center
4200 East 9th Ave.
Denver, Colorado 80262

